# HAART restores the frequency of regulatory T cells (Tregs) among HIV/AIDS patients

**DOI:** 10.1186/1471-2334-14-S3-E1

**Published:** 2014-05-27

**Authors:** M Suthandhira, H Durgadevi, OR Krishnarajasekar, K Raja, B Rayvathy, Sowmya Swaminathan, Luke Elizabeth Hanna, S Anbalagan, Elanchezhiyan Manickan

**Affiliations:** 1 Department of Microbiology, Dr. ALM PG IBMS, University of Madras, Taramani, Chennai, India; 2 Govt. Hospital of Thoracic Medicine, Tambaram Sanatorium, Chennai, India; 3 National Institute for Research in Tuberculosis (ICMR), Chetpet, Chennai, India

## Background

Immunosuppression is the hallmark HIV infection associated with gradual loss of CD4+Tcells. The effector immune response is regulated by CD4+CD25+Foxp3+Tcells (Tregs). Enumeration of Tregs frequency showed equivocal results before and here we showed clearly their frequency by flowcytometry.

## Methods

One hundred and eight individuals comprising of 71 HIV positive patients (44 patients undergoing HAART and 27 without HAART) and 37 HIV negative healthy controls. PBMCs were isolated and stained with CD4/FITC, CD25/APC, FoxP3/PE antibodies (BD Pharmingen) and analyzed for Tregs frequency by flowcytometer (BD FACS Calibur).

## Results

Our study showed that healthy controls had a Tregs frequency of 4.1%. When Tregs frequency analyzed among HIV/AIDS patients who are yet to receive HAART we found a significant reduction in Tregs population i.e. 1.2%. Encouragingly this deep plunge in Tregs population was found to be restored and surged upon HAART up to 12.6%. However, these changes in percentages did not alter based on their gender or age or absolute CD4 count of the study population.

## Conclusion

HAART is known to decrease viral load and improve the patient’s CD4 cells count. Here we report that HAART has improved the Tregs frequency. It is yet to be known that the consequence of resurging Tregs during HIV/AIDS.

